# *Streptococcus salivarius* MS-oral-D6 promotes gingival re-epithelialization *in vitro* through a secreted serine protease

**DOI:** 10.1038/s41598-017-11446-z

**Published:** 2017-09-11

**Authors:** Marcela M. Fernandez-Gutierrez, Peter P. J. Roosjen, Eveline Ultee, Maarten Agelink, Jacques J. M. Vervoort, Bart Keijser, Jerry M. Wells, Michiel Kleerebezem

**Affiliations:** 1 TI Food and Nutrition, Nieuwe Kanaal 9-A, 6709 PA Wageningen, The Netherlands; 2 Host-Microbe Interactomics Group, Department of Animal Sciences, Wageningen University & Research, De Elst 1, 6708 WD Wageningen, The Netherlands; 3 Laboratory of Geo-Information Science and Remote Sensing, Wageningen University & Research, Droevendaalsesteeg 3, 6708 PB Wageningen, The Netherlands; 4 Biochemistry Group, Department of Agrotechnology and Food Sciences, Wageningen University & Research, Stippeneng 4, 6708 WE Wageningen, The Netherlands; 5 TNO Microbiology and Systems Biology, Utrechtseweg 48, 3704 HE Zeist, The Netherlands; 6 Department of Preventive Dentistry, Academic Centre for Dentistry Amsterdam, University of Amsterdam and Vrije Universiteit Amsterdam, Gustav Mahlerlaan 3004, 1081 LA Amsterdam, The Netherlands

## Abstract

Gingival re-epithelialization represents an essential phase of oral wound healing in which epithelial integrity is re-establish. We developed an automated high-throughput re-epithelialization kinetic model, using the gingival epithelial cell line Ca9–22. The model was employed to screen 39 lactic acid bacteria, predominantly including oral isolates, for their capacity to accelerate gingival re-epithelialization. This screen identified several strains of *Streptococcus salivarius* that stimulated re-epithelialization. Further analysis revealed that *S. salivarius* strain MS-oral-D6 significantly promoted re-epithelialization through a secreted proteinaceous compound and subsequent experiments identified a secreted serine protease as the most likely candidate to be involved in re-epithelialization stimulation. The identification of bacteria or their products that stimulate gingival wound repair may inspire novel strategies for the maintenance of oral health.

## Introduction

Certain members of the lactic acid bacteria (LAB), in particular the lactobacilli, have been widely used as probiotics in animal feeds and human products as they are generally recognized as safe (GRAS status) by The American Food and Drug Administration (FDA). Probiotics are “live microorganisms that when administered in adequate amounts, confer a health benefit on the host”^1^. Probiotics have been reported to exert numerous beneficial effects on gut health, for example by antagonizing the growth of pathogenic bacteria^2^, promoting host-microbe homeostasis through modulation of immunity^3, 4^, alleviating symptoms of lactose intolerance^5^, enhancing mucosal barrier function^6–8^, and promoting intestinal epithelial survival and growth^9, 10^. Currently, there is an increasing interest in exploring the beneficial health effects of these probiotic bacteria in other body-sites such as the skin^11^, the urogenital tract^12, 13^ and the oral cavity^14^. Studies on the beneficial effects of LAB in the oral cavity have explored their ability to persist in this niche^15, 16^, their antimicrobial activity against cariogenic and periodontal pathogens^17, 18^, their capacity to diminish malodour^19^, and their ability to promote host-microbe homeostasis^20^. However, to our knowledge, there are no studies reporting beneficial effects on gingival wound healing.

The oral gingiva consists of stratified squamous epithelium that serves as a barrier between the external environment and the underlying tissue. Upon disruption of the barrier integrity, the processes involved in clearance of infection and renewal of damaged cells are immediately initiated. Healing of acute wounds occurs in four overlapping phases: clot formation, inflammation, re-epithelialization, and tissue remodelling^21–24^, regulated by a complex signalling network that involves numerous growth factors, cytokines and chemokines. Wounds that fail to proceed through the normal stages of wound repair in a timely and orchestrated manner result in chronic wounds, which are often colonized by bacteria that may contribute to the delayed or incomplete healing process by perpetuating inflammatory responses. This is the case in periodontitis, a bacterial infection characterized by chronic inflammation of the periodontal tissue that ultimately leads to destruction of the connective tissue and subsequent bone and tooth loss^25, 26^. Prompt re-epithelialization is considered as one of the key parameters for optimal wound repair and prevention of chronic infections^27^. Re-epithelialization is driven by the proliferation and migration of epithelial cells into the site of injury to re-establish cell-to-cell contacts and close the wound. Scratch assays have been widely used as an *in vitro* model to study the influence of particular compounds on re-epithelialization. Such assays consist of the mechanical introduction of a scratch (“wound”) into a confluent epithelial cell monolayer and following the re-epithelialization of that scratch by the acquisition of microscopic images over time^28^. However, these assays tend to be executed at low throughput and in general suffer from poor reproducibility^29^ and absence of real-time kinetic data. Here we describe the development of a high-throughput scratch assay using live-automated fluorescence microscopy, image segmentation, and quantitative data processing to model the kinetics of re-epithelialization of the gingival epithelial cell line Ca9–22 (JCRB0625). Using this assay, we screened 39 strains of LAB for their potential effects on re-epithelialization, hypothesising that certain LAB strains produce specific compounds that can accelerate epithelial wound repair. The primary screening results were extended and confirmed in subsequent refined experiments that enabled the identification of *Streptococcus salivarius* MS-oral-D6 and its secreted protease as strong stimulators of gingival re-epithelialization.

## Results

### A novel strategy to extract and quantify the kinetics of the re-epithelialization process

**Figure 1:**
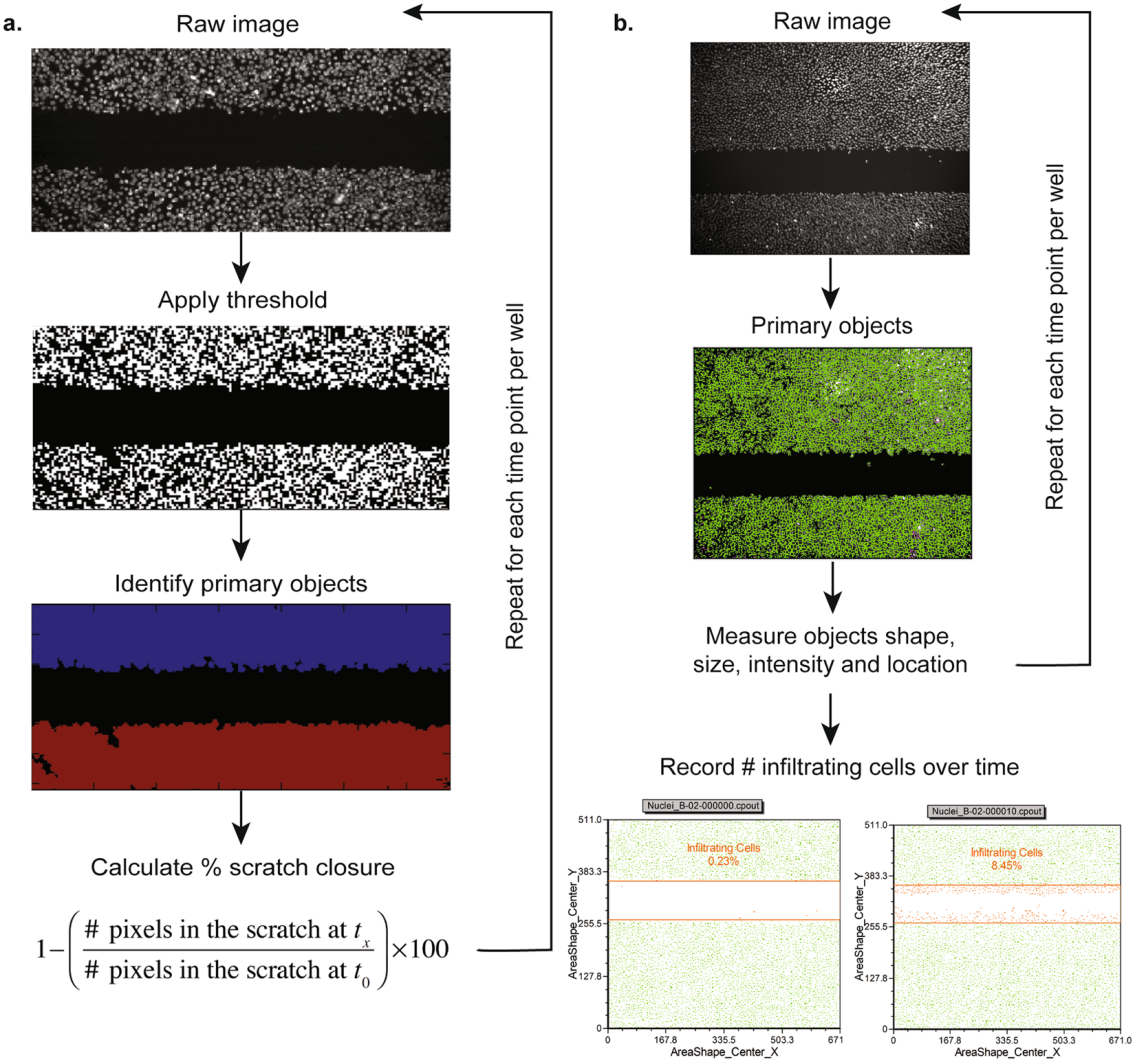
Automated image segmentation pipelines. (**a**) The tissue-recognition pipeline identifies the monolayer of cells as objects and the remaining pixels as the scratch. Thereafter, the percentage of scratch closure in consecutive images can then be calculated. (**b**) The cell-recognition pipeline identifies single cells in sequential images and records their location. A gate is manually set on the scratched area at time 0 and the number of epithelial cells infiltrating into the gate are recorded over time.

The throughput and reproducibility of the scratch assay was improved in this study by using a 96-well system and the HTSScratcher, which consists of a 96 pin-array controlled by a counterforce mechanism that ensured that the downward movement of the array towards the 96-well plate was conducted smoothly and consistently. Furthermore, we were able to quantify re-epithelialization over time by using automated microscopy in combination with image analysis. Images were acquired every 20 minutes until the scratches treated with human transforming growth factor α (hTGFα; positive control) were fully resolved. The tissue-recognition pipeline (Fig. 1a) of image analysis required a fully confluent cell monolayer to accurately determine the scratch size, but resembled the conventional way of analysing scratch assays in which the closure achieved with different treatments is compared after fixed periods of time^28^. The cell-recognition pipeline (Fig. 1b) enabled effective extraction of quantitative data by automatic identification of the number of cells in each image, which was accompanied by information regarding size, shape, stain-intensity, and location of each cell. The data acquired through this pipeline was visualized using the dedicated software FCS Express 4 Plus (De Novo Software, CA, USA) in which the number of cells infiltrating into the scratched area over time was determined for each well.

**Figure 2:**
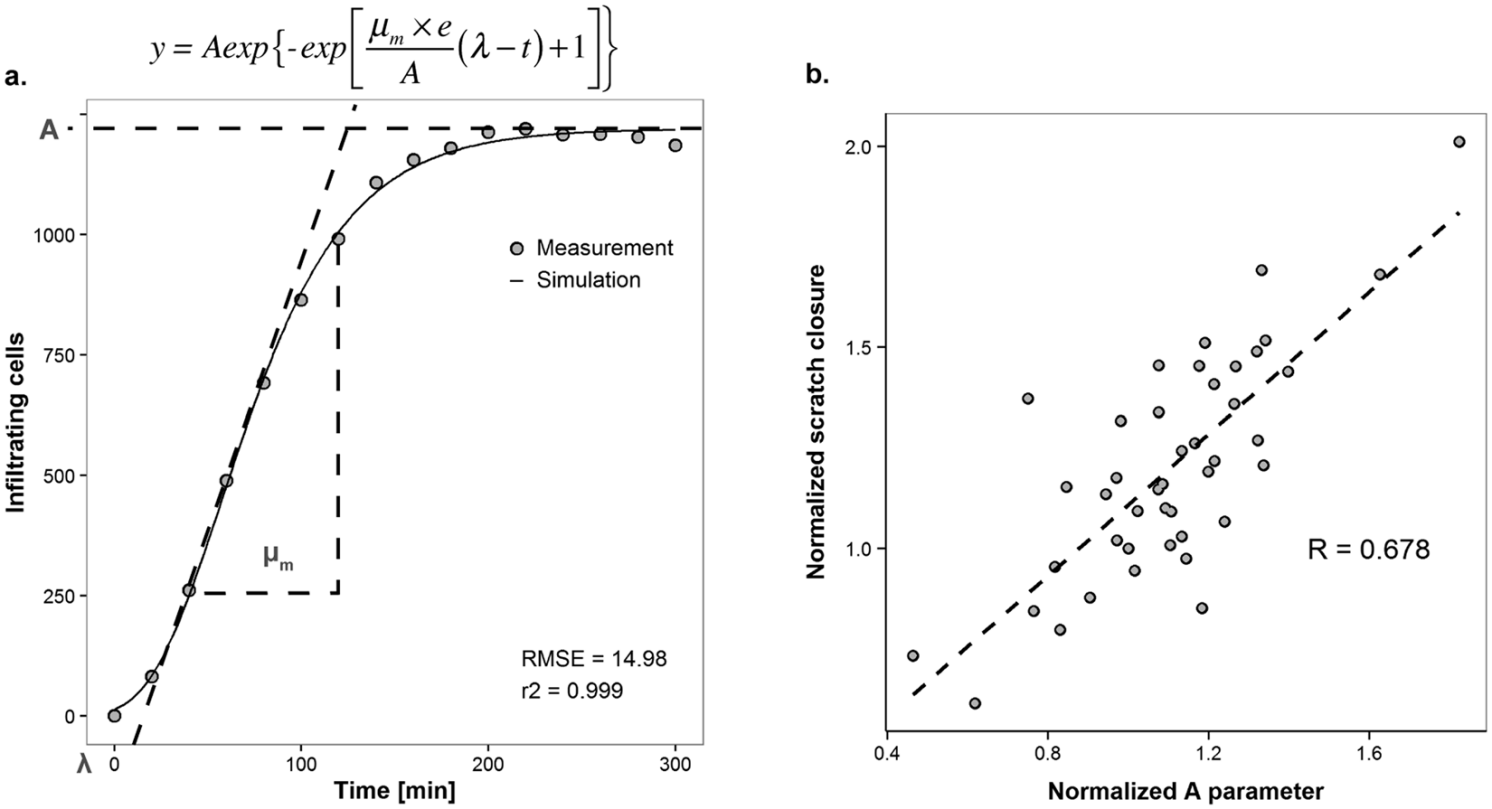
Modelling gingival re-epithelialization. (**a**) Representative sigmoidal curve obtained from the enumeration of epithelial cells infiltrating into the scratched area over time. Modified Gompertz function for sigmoidal curve fitting consisting of three parameters: the inflection point (λ, in minutes), the repair rate (μ_m_, in cells minute^−1^) and the maximum number of cells (A, in cells). (**b**) Spearman Rank Correlation coefficient to assess the relation between the A parameter and the relative scratch closure. All values were normalized against the non-treated control.

The enumeration of infiltrating cells over time consistently resulted in a sigmoidal curve. Therefore, the modified Gompertz function (Fig. 2a), which has previously been used to model bacterial growth curves^30^, was fitted through the re-epithelialization measurements by a nonlinear least squares regression to estimate three biologically comprehensive parameters: μ_m_, A and λ. The μ_m_ parameter represents the repair rate (cells minute^−1^), and the A parameter represents the maximum number of cells within the scratched area at the plateau phase of the growth curve. The lag time or inflection point (λ, described in minutes) represents the amount of time required for the cells to start moving into the scratched area and typically ranged from negative values to 20 minutes (Supplementary Table S1). This implies that Ca9–22 cell- migration was already active when the image acquisition was initiated, and indicates that although the calculation of the λ parameter is required to obtain a good fit of the model, its biological value was negligible. Thereby, the μ_m_ and A parameters were sufficient to accurately describe the re-epithelialization kinetics with this cell line. Overall, the model yielded very good fits (Supplementary Table S1; R^2^ close to 1), illustrating that it effectively extracts quantitative re-epithelialization parameters from the serial images obtained and providing a high-throughput platform for quantification of wound repair kinetics. Moreover, the maximum number of cells (A parameter) obtained with the model was highly correlated with the relative scratch closure measured using the tissue-recognition pipeline (r = 0.678, *P* = 1.36 × 10^−6^), which corroborated that enumeration of cells that infiltrated the scratched area provides a parameter that reflects the relative closure values obtained through the tissue-recognition pipeline (Fig. 2b). The workflow and crucial steps in the overall procedure are illustrated in Supplementary Fig. S1.

### Selection of potential stimulators and attenuators of gingival re-epithelialization

**Figure 3:**
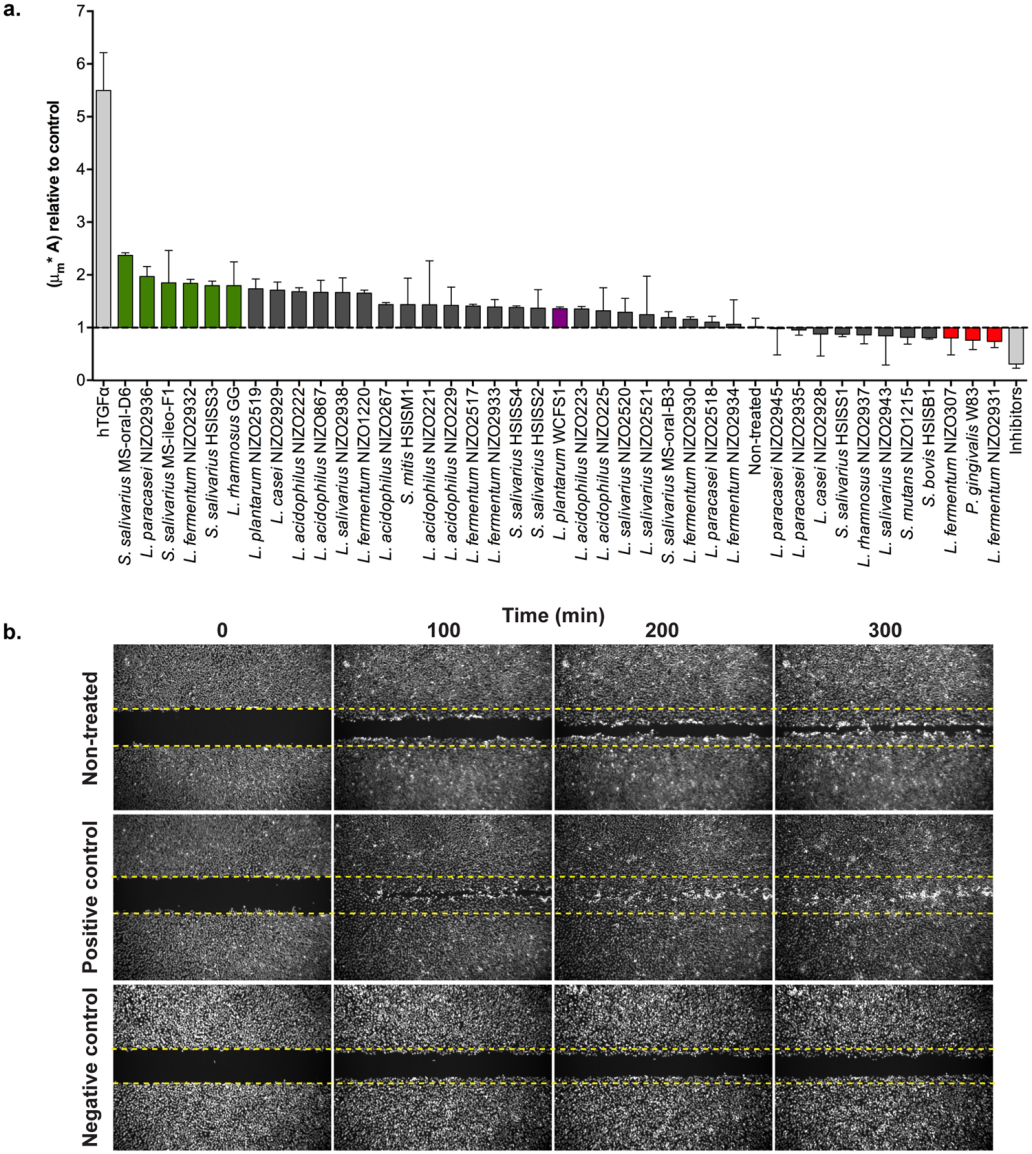
Modulatory effect of lactic acid bacteria in gingival re-epithelialization. (**a**) Overall performance of the bacterial treatments relative to the non-treated control (μ_m_*A). Results are expressed as mean ± SEM. Green bars: potential stimulators; red bars: potential attenuators; purple bar: minor modulator. (**b**) Representative images for the non-treated, positive (hTGFα, 4 ng/μl) and negative (p38 and MEK1/2 inhibitors, 10 μM each) controls.

The established scratch assay and downstream analysis method were used to screen a panel of 39 lactic acid bacteria (LAB) (Table 2) for their capacity to modulate the kinetics of gingival re-epithelialization. The initial screening was performed using duplicate measurements, and employing the Z’ factor^31^ to assess the quality of individual assay runs on basis of the calculated dynamic range between of the positive and negative controls. Stimulation of the gingival cells with the hTGFα increased re-epithelialization kinetics more than 5-fold, and the combination of p38 and MEK1/2 inhibitors suppressed re-epithelialization kinetics approximately 2-fold, as compared to the non-treated control condition, respectively (Fig. 3).

Most of the bacterial strains tested were originally isolated from the oral cavity. Other typical residents of the oral cavity (i.e. *Streptococcus* strains) were isolated from human ileostoma effluent, but were proposed by the authors^32^ to have originated from the oral cavity. In order to select potential stimulators and attenuators of re-epithelialization kinetics, a combinatorial approach was chosen that incorporated both relevant kinetic parameters from the measurements (μ_m_ and A) into a single performance value (μ_m_*A) that was normalized against the non-treated control in each independent assay run (Fig. 3). Importantly, the cell-based assay and the automated pipelines of analysis clearly confirmed the previously reported stimulating effect of *L. rhamnosus* GG^10, 11, 33^ and the suppressive effect of *P. gingivalis* ^33, 34^ on re-epithelialization, which were both included in this study as reference strains for the establishment of the assay (Fig. 3; see also Supplementary Fig. S2). Exposure of the gingival cells to LAB at a multiplicity of infection (MOI) of 50 noticeably resulted in strain-specific modulatory effects of gingival re-epithelialization with some members of the same species having opposing effects (e.g. *S. salivarius* MS-oral-D6 and *S. salivarius* HSISS1; *L. fermentum* NIZO2932 and *L. fermentum* NIZO2931). Overall, the results showed a common tendency of LAB to enhance the healing process, but none to a level that was comparable to the hTGFα-stimulated cells (Fig. 3a). The bacteria that elicited the strongest modulatory effects were selected for further studies. *S. salivarius* MS-oral-D6 and *L. paracasei* NIZO2936 were identified as the most prominent stimulators of gingival re-epithelialization, enhancing its kinetics by approximately 2.5 and 2-fold relative to the non-treated control, respectively. The selection of potential stimulators of gingival re-epithelialization was expanded with the next four strongest modulators of the process namely, *S. salivarius* MS-ileo-F1, *L. fermentum* NIZO2932, *S. salivarius* HSISS3 and *L. rhamnosus* GG (Fig. 3a). Furthermore, we were also capable of detecting potential suppressors of epithelial repair in comparison to the non-treated control. To this end, *L. fermentum* NIZO2931 and *L. fermentum* NIZO307 were selected for further studies as they appeared to inhibit re-epithelialization to a level that was comparable to *P. gingivalis* W83. Finally, *L. plantarum* WCFS1, which is among the most intensely studied *Lactobacillus* strains, was selected as a representative of the strains that were more or less ineffective modulators of gingival re-epithelialization.

### Dose-response analysis confirms modulatory effects of selected bacteria on gingival re-epithelialization

**Figure 4:**
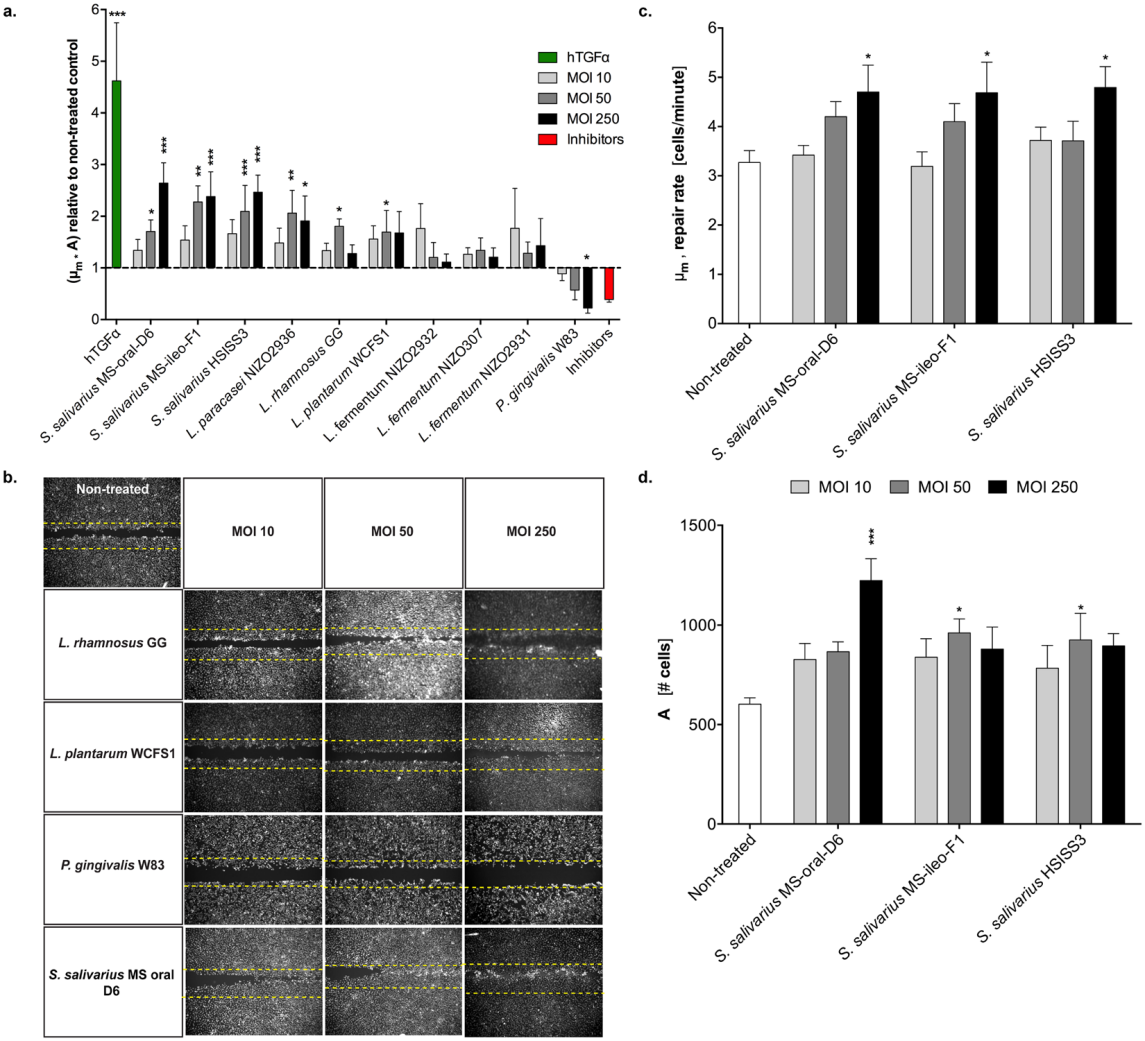
Dose response analysis of selected bacteria on gingival re-epithelialization. (**a**) The performance value (μ_m_ * A) of each treatment was calculated relative to the non-treated control. (**b**) Representative images of the re-epithelialization process after 5 hours exposure to bacteria at a multiplicity of infection (MOI) of 10, 50 and 250. (**c**) Repair rate obtained for the *Streptococci* strains. (**d**) Maximum number of cells in the scratched area obtained for the *Streptococci* strains. Results are expressed as mean ± SEM from at least two independent experiments performed in triplicates. Significant differences from the non-treated control were assessed by a one-way ANOVA using a Dunnett’s test for multiple comparisons (*P < 0.05; **P < 0.001; ***P < 0.0001).

In order to confirm and refine the modulatory effects of the selected bacteria on gingival re-epithelialization, the scratch assay was performed using increasing dosages of bacterial preparations (MOI of 10, 50 and 250) and a higher number of replicates (n = 3) in at least two independent experiments. Notably, all *S. salivarius* strains selected improved the re-epithelialization performance (μ_m_*A) in a dose-dependent manner, resulting in a significant increase at the intermediate and higher dosages (*S. salivarius* MS-oral-D6 MOI 50 (*P* = 0.039) and 250 (*P* < 0.0001); *S. salivarius* MS-ileo-F1 MOI 50 (*P* < 0.0001) and 250 (*P* < 0.0001); *S. salivarius* HSISS3 MOI 50 (*P* = 0.001) and 250 (*P* < 0.0001)) (Fig. 4a). Specifically, the *S. salivarius* strains accelerated the repair rate (μ_m_ parameter) in a dose dependent manner, leading to a higher number of cells (A parameter) in the scratched area as compared to the non-treated control (Fig. 4c,d). The promoting effect of *L. rhamnosus* GG on the scratch resolution was also confirmed for the intermediate MOI tested (*P* = 0.011) (Fig. 4a,b), but it failed to significantly enhance the re-epithelialization kinetics at a higher dosage. Similarly, both the intermediate and higher dosages of *L. paracasei* NIZO2936 promoted re-epithelialization by approximately 2-fold and thus failed to show a clear dose-response relationship. Treatment with *L. plantarum* WCFS1 resulted in minor modulation of the re-epithelialization process regardless of the dosage used, confirming the lack of modulatory capacity of this strain in the re-epithelialization process as was concluded from the initial screening experiment (Fig. 4a,b). Exposure to increasing dosages of *P. gingivalis* W83 resulted in significant inhibition of re-epithelialization (*P* = 0.037), ultimately leading to actual deterioration of the cell monolayer (Fig. 4a,b). Finally, contrary to the initial screening results, stimulation of the epithelial cells with different dosages of the *L. fermentum* strains NIZO2931, NIZO2932 and NIZO307 did not result in significant modulation of the healing process (Fig. 4a).

### Influence of bacterial fermentation products and inflammatory capacity on re-epithelization

**Figure 5:**
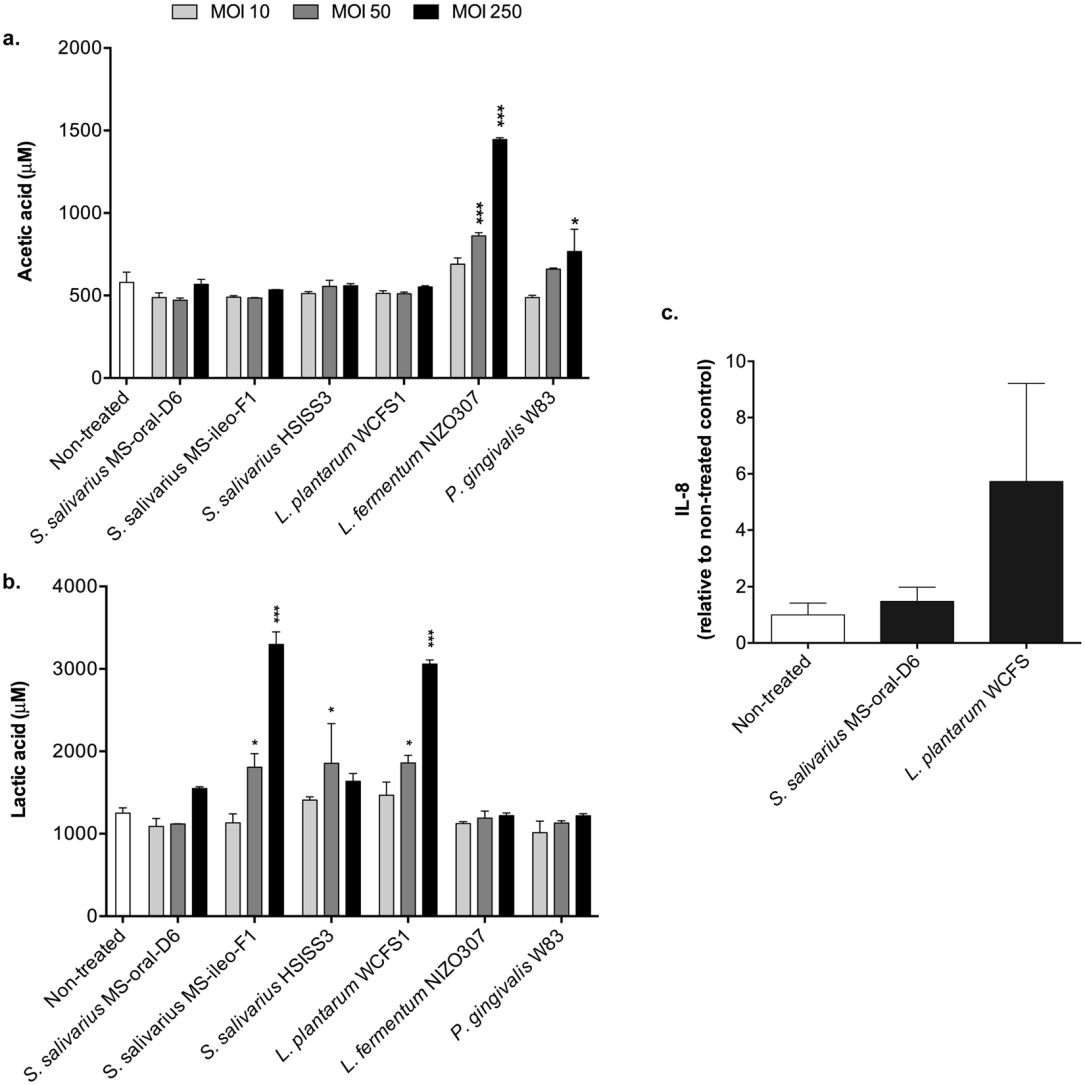
Fermentation end products of bacteria and gingival inflammatory response. (**a**) Acetic acid. (**b**) Lactic acid. The concentrations of these end-products of bacterial fermentation were measured in the supernatants of gingival cells stimulated with increasing MOIs of bacteria after 5 hours. Results are expressed as mean ± SEM from two independent experiments performed in triplicates. Significant differences from the non-treated control were assessed by a one-way ANOVA using a Dunnett’s test for multiple comparisons (*P < 0.05; ***P < 0.0001). (**c**) Interleukin-8 (IL-8) produced by the gingival cells after 5 hours of exposure to the bacteria (MOI 250) expressed relative to the non-treated control. Results are expressed as mean ± SEM from two independent experiments performed in duplicates.

To evaluate whether the modulatory effects of some bacteria on the kinetics of re-epithelialization were the result of the accumulation of fermentation end products, we quantified the concentration of lactic and acetic acid in the supernatants of the epithelial cell cultures 5 hours after addition of different dosages of bacteria (Supplementary Table S2). The putative correlation between the detected concentrations of the lactic acid and acetic acid in individual wells with the kinetic parameter values (μ_m_ and A) was subsequently evaluated by Pearson correlation analysis. A weak, but significant negative correlation (r = −0.279, *P* = 0.04) between the acetic acid concentration and the A parameter was detected (Supplementary Fig. S3). Notably, the concentrations of acetic acid in the supernatants of the cells exposed to the higher dosages of *L. fermentum* NIZO307 and *P. gingivalis* W83, two of the strains with negative modulatory effect on re-epithelialization, were significantly higher than those of the non-treated control (*P* < 0.0001 and *P* = 0.012, respectively) (Fig. 5a). Conversely, significant positive correlations were detected between the lactic acid concentration and repair rate (μ_m_; r = 0.378, *P* = 0.004) and the maximum number of cells (A; r = 0.354, *P* = 0.008). The concentrations of lactic acid in the supernatants of cells exposed to *S. salivarius* MS-ileo-F1 (MOI 50, *P* = 0.019; MOI 250, *P* < 0.0001) and *S. salivarius* HSISS3 (MOI 50, *P* < 0.009) contained significantly more lactic acid than the non-treated control and were among the positive modulators of re-epithelialization kinetics. However, the strongest positive modulator of gingival re-epithelialization, *S. salivarius* MS-oral-D6 (Fig. 4), did not produce significant amounts of lactic acid during the assay, whereas *L. plantarum* WCFS1 produced substantial amounts of lactic acid (>3 mM) and failed to enhance the re-epithelialization process (Fig. 5b), indicating that these bacteria can influence the repair process via different mechanisms. Notably, exposure of the Ca9–22 cell line to *L. plantarum* WCFS1, but not to *S. salivarius* MS-oral-D6 (both at MOI 250), elicited considerable production of the pro-inflammatory cytokine interleukin-8 (IL-8) (Fig. 5c), which may counteract the potentially stimulatory effect on re-epithelialization of the lactic acid produced by *L. plantarum* WCFS1.

### The secreted serine protease of *S. salivarius* MS-oral-D6 accelerates gingival re-epithelialization

**Figure 6:**
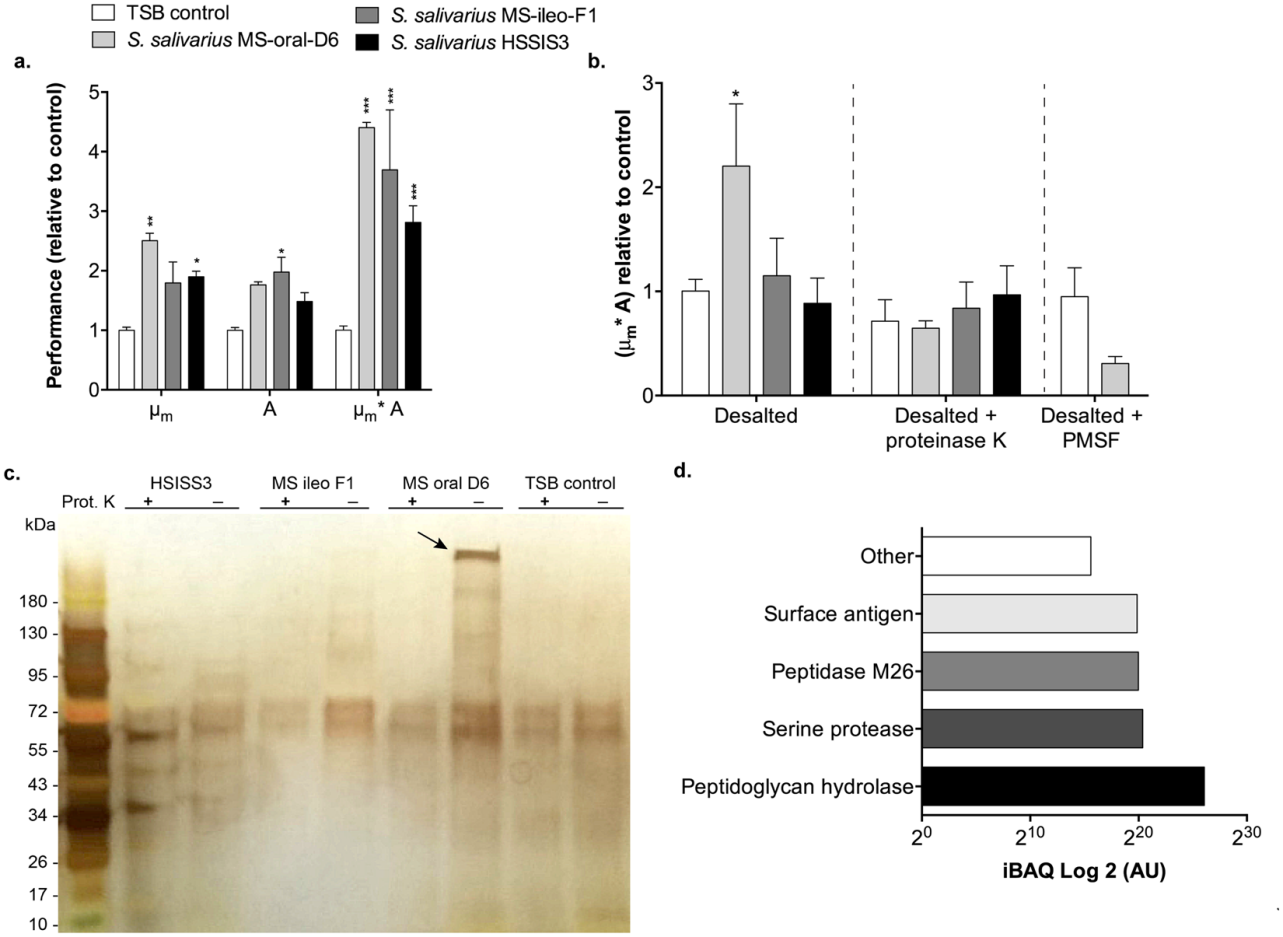
Evaluation of conditioned medium of *Streptococcus salivarius* strains on gingival re-epithelialization. (**a**) Re-epithelialization parameters (μ_m_, A, and μ_m_*A) of the *S. salivarius* conditioned medium relative to the TSB control. (**b**) Re-epithelialization performance values obtained with desalted-conditioned medium (7 kDa cut-off) with or without proteinase K treatment (200 μg/ml, 1 hour at 37 °C). *S. salivarius* MS-oral-D6 desalted-conditioned medium after PMSF treatment (1 mM, 1 hour at room temperature). Results are expressed as mean ± SEM from two independent experiments performed in triplicates. Significant differences from the non-treated control were assessed by a one-way ANOVA using a Dunnett’s test for multiple comparisons (*P < 0.05; **P < 0.001; ***P < 0.0001). (**c**) Silver staining of the proteins present in desalted-conditioned medium before and after proteinase K (Prot. K) treatment. Arrow indicates the high MW band detected in *S. salivarius* MS-oral-D6 conditioned medium. (**d**) Abundant proteins identified in the high MW band of the *S. salivarius* MS-oral-D6 conditioned medium. The bar labelled as “other” represents the sum abundance of seven other identified secreted proteins. Intensity based absolute quantitation (iBAQ) refers to the total intensity corrected for the number of measurable peptides within a sample.

To determine whether the stimulation of gingival re-epithelialization by *S. salivarius* strains was due to an extracellular molecule we tested spent culture supernatant (conditioned medium) in the scratch assay. The conditioned media of the *S. salivarius* strains tested were consistently accelerating re-epithelialization kinetics (μ_m_, A, and μ_m_*A) when compared with the original medium (i.e., TSB) (Fig. 6a), indicating that these bacteria may indeed secrete a component that is capable of promoting wound healing. To further investigate these effects, we removed small molecules and peptides from the conditioned medium using desalting columns with a 7 kDa cut-off. The stimulatory effects of conditioned medium from *S. salivarius* MS-ileo-F1 and HSISS3 on re-epithelialization were lost after passing the medium over a desalting column (Fig. 6b), suggesting that the activity observed was due to the presence of small molecules (<7 kDa), possibly involving lactic acid. In contrast, the conditioned medium of *S. salivarius* MS-oral-D6 retained its stimulatory effect on re-epithelialization after desalting, which was subsequently shown to be sensitive to proteinase K treatment (200 μg/ml), indicating that the activity is due to a secreted protein (Fig. 6b). In addition, silver stained SDS-PAGE analysis of the desalted-conditioned medium of the *S. salivarius* strains revealed the presence of a high molecular weight (MW) protein (>180 kDa) in the medium of *S. salivarius* MS-oral-D6 that was completely digested after proteinase K treatment (Fig. 6c) and was absent from the media obtained from *S. salivarius* MS-ileo-F1 and HSISS3. In-gel trypsin digestion of the high MW protein followed by liquid chromatography tandem mass spectrometry (LC-MS/MS) and peptide mapping against the available *S. salivarius* genomes sequences (37 in total; Supplementary Table S3) revealed the presence of 11 proteins from which the most abundant were identified as peptidoglycan hydrolase, serine protease, peptidase M26, and surface antigen (Fig. 6c and Table 1). From the identified proteins only the serine protease and the peptidase M26 have a predicted MW that match the apparent MW of the band observed in the gel of >180 kDa (Table 1). Importantly, the gene encoding the serine protease appears to be encoded by only a fraction of the known *S. salivarius* genome sequences (12 out of 37), whereas the three other proteins appeared to be encoded by the vast majority of *S. salivarius* genomes (including the HSISS3 strain) (Table 1), which may support a role of the serine protease in the strain-specific stimulatory effect observed. Finally, to further investigate the possible involvement of the serine protease in enhanced re-epithelialization, the desalted-conditioned medium of *S. salivarius* MS-oral-D6 was pretreated with the serine protease inhibitor phenylmethane sulfonyl fluoride (PMSF, 1 mM) prior to being tested in the scratch assay. Following PMSF inactivation, addition of the MS-oral-D6 desalted-conditioned medium led to a reduction in  re-epithelialization kinetics when compared to the control (Fig. 6c), supporting that the secreted serine protease of *S. salivarius* MS-oral-D6 is the effector molecule responsible for the enhanced re-epithelialization kinetics observed.

**Table 1:** Proteins identified in the conditioned medium of *S. salivarius* MS-oral-D6.

Protein	Accession	Gene ID	Size (kDa)	Presence (%) sequenced genomes
Peptidoglycan hydrolase	A0A0A1DSA9	SSAL8618_00185	56	92
Serine protease	A0A074J261	DL07_03090	241	32
Peptidase M26	A0A074IYQ3	DL07_02275	222	81
Surface antigen	F8HFL8	Ssal_01709	114	92

UniProt accession entries and gene IDs are provided with the predicted protein sizes (kDa). The presence of the respective proteins in the sequenced genomes of *S. salivarius* is indicated as a percentage of the total number of genomes available (n = 37).

## Discussion

Lactic acid bacteria (LAB), in particular lactobacilli and streptococci, have been generally associated with caries^34, 35^. Therefore, the idea of applying these microorganisms to promote oral health seems controversial. Nevertheless, there is increasing evidence implicating specific LAB in oral health rather than disease. For instance, *L. plantarum* 299v, *L. reuteri* (ATCC 55730 and ATCC PTA 5289) and *L. rhamnosus* GG have been shown to interfere with biofilm formation of *S. mutans in vitro*, which is still regarded as the primary etiological agent of dental caries^36^. Similarly, several isolates of *L. paracasei* and *L. rhamnosus* derived from healthy oral cavities, were shown to have antimicrobial activity against *S. mutans* and other oral pathogens including *P. gingivalis* and *C. albicans* ^17^. Clinical interventions have provided evidence that *L. reuteri* ATCC 55730 can suppress cariogenic bacteria^37–39^ and improve periodontal health by diminishing gingival bleeding and reducing the levels of proinflammatory cytokines in subjects with moderate gingival inflammation^40^. In other studies, administration of *L. salivarius* WB21 or *S. salivarius* K12 resulted in improved periodontal health at the end of an intervention^41, 42^ and suppression of volatile sulphur compounds (VSC)-producing bacteria, which are responsible for halitosis or “bad breath”^19, 42^.

In this study, we evaluated the ability of oral LAB to promote gingival epithelial repair. The oral epithelium is the first line of defence against invading microorganisms and thus, upon disruption of barrier integrity, prompt re-epithelialization is essential^43, 44^. To study the effects of different LAB on re-epithelialization, we developed a high-throughput scratch assay using live-automated fluorescence microscopy and image segmentation, which is coupled to a mathematical model that describes the kinetics of re-epithelialization. Contrary to traditional scratch assays, which are executed in 24-well plates and rely upon the manual introduction of a scratch^28^, we increased the throughput and reproducibility of the scratches by changing the assay format to 96-well plates and using the HTSScratcher (Peira) to generate uniform scratches. Previous studies^45, 46^ have also been able to increase the throughput of the scratch assay by employing different scratching tools such as a 96-pin floating array and an 8-channel “wounder”. However, these tools lacked the counterforce mechanism of the HTSScratcher, which ensures a smooth and consistent movement of the pin-array parallel to the wells. Consequently, HTSScratcher generates uniformly sized scratches with minimal damage to the plate coating, which could otherwise interfere with normal cell migration.

A key aspect of this high-throughput image-based method is the ability to automate the conversion of raw images into interpretable data. Hence, we established two analysis pipelines for image analysis in CellProfiler, one based on tissue-recognition and the other on cell-recognition. The tissue-recognition pipeline is a robust and straightforward analysis to determine the percentage of scratch closure through the image series of each well. Other open-source software such as ImageJ (https://imagej.nih.gov/ij/; “MRI Wound Healing Tool”) and TScratch^47^ provide alternative options to carry out this type of analysis. Nevertheless, enumeration of single cells migrating into the scratched area over time provides quantitative data that can more readily be statistically analysed and enables the possibility of single cell tracking. Re-epithelialization kinetics were described by biologically comprehensive parameters (μ_m_, A and λ) derived from the modified Gompertz function^30^. The applied iterative optimization method yielded excellent fits and provided accurate estimation of the kinetics of the wound healing process.

Several studies have reported that *Porphyromonas gingivalis* attenuates re-epithelialization in scratch assays employing cell lines or primary gingival cells, and have proposed that this effect was at least partially dependent on the proteolytic activity secreted by this bacterial species^48, 49^. Conversely, *Lactobacillus rhamnosus* GG has been shown to influence intestinal epithelial cells through the secretion of proteins (p75 and p40) that interact with the epidermal growth factor receptor (EGFR) to promote cell proliferation, differentiation, migration, and survival^9, 50^. More recently, *L. rhamnosus* GG lysates were reported to promote re-epithelialization of skin-keratinocytes by increasing cell migration *in vitro* ^11^, highlighting the possibility of expanding the applications of certain strains to promote beneficial effects in other body-sites such as the skin and the oral cavity. Hence, we included these microorganisms as benchmark bacteria to validate the assay developed in this study. The results obtained with these strains clearly confirm the attenuating and stimulating effects of *P. gingivalis* and *L. rhamnosus* GG in the Ca9–22 gingival re-epithelialization model, respectively.

The results of the preliminary screening revealed the common tendency of LAB to stimulate re-epithelialization kinetics, although none of the strains tested stimulated to a level comparable to the positive control (hTGFα). The extent of the modulatory effect exerted by the bacteria could have been a result of the dosage selected for the screening (50 bacterial cells per epithelial cell; MOI 50) and/or the administration of viable bacteria. Interestingly, Mohammedsaeed *et al*.^11^ showed that *L. rhamnosus* GG lysates could promote skin-keratinocyte re-epithelialization to a level comparable to the keratinocyte growth factor used as positive control in that study. However, the lysate dosage employed in the study was very high (93 mg/well) and exceeded the highest corresponding dosage of viable cells employed here by at least 1,000-fold. Dosages higher than the highest dose employed in this study (MOI 250) of viable *L. rhamnosus* GG (or many other LAB) are not feasible due to their capacity to acidify the cell culture medium during the assay, which interferes with cell viability. The initial screening revealed strain-specific effects on re-epithelialization by the different LAB, where *L. fermentum* NIZO2932 was identified as a potential stimulator, and *L. fermentum* NIZO2931 and NIZO307 seemed to have the opposite effect. Nevertheless, upon exposure of the epithelial cells to increased dosages of these bacteria, a weakly negative effect on re-epithelialization was observed for all *L. fermentum* strains. Notably, high-dosages of lysates of *L. fermentum* ATCC 14932 (NIZO2930) were previously reported to significantly reduce skin-keratinocyte re-epithelialization *in vitro* ^11^, but substantially lower dosages of viable cells of the same strain did not show modulatory effect in our model (Fig. 3a), which again may relate to the dosages used in the different studies.

Among the strongest and most consistent stimulators of oral re-epithelialization were the *Streptococcus salivarius* strains MS-oral-D6, MS-ileo-F1 and HSISS3. *S. salivarius* is one of the first bacterial species to colonize the intestinal and oral epithelial mucosa after birth^51^ and remains as a relatively dominant inhabitant of the oral cavity, upper respiratory tract and small intestine of healthy individuals throughout life^52–54^. Remarkably, co-culture of human bronchial epithelial cells with probiotic *S. salivairus* K12 altered the expression of 565 host genes involved in innate immunity pathways, epithelial cell function, cytoskeletal remodelling, cell development and migration^20^. Exposure of gingival epithelial cells to *S. salivarius* strains in this study resulted in acceleration of the repair rate in a dose-dependent manner. Although our results indicated that the re-epithelialization kinetics could be affected by the production of the main fermentation end products of these LAB (i.e., acetic and lactic acid), these metabolites could not explain the stimulatory effects of the *S. salivarius* strains. In particular, *S. salivarius* MS-oral-D6 did not produce significant amounts of either of these metabolites and was the strongest stimulator of re-epithelialization. Previous *in vivo* wound-healing studies have shown that lactate accumulates in wounds (4–12 mM) and regulates the healing process by stimulating the expression of the vascular endothelial growth factor (VEGF) and collagen deposition^55, 56^. However, the concentrations of lactic acid measured after 5 hours in the culture media that contained LAB were in most cases lower than 4 mM. An exception was the cells exposed to the highest dosage of *L. paracasei* NIZO2936, where lactic acid concentrations increased to final concentrations of up to 5 mM (Supplementary Table S2) and only moderate stimulation of re-epithelialization kinetics was observed. These findings suggest that the contribution of the lactic acid to the wound-healing process in our study was limited, and indeed concentrations up to 6 mM of D- or L-lactic acid at neutral pH did not affect the re-epithelialization kinetics in our model (Supplementary Fig. S4), implying that other components play an important role in the observed modulations of re-epithelialization kinetics. Among such other components, may be those that trigger pro-inflammatory responses in epithelial cells, which may delay wound repair by blocking the essential transition from the inflammatory to the proliferative phase of wound healing^57, 58^. Measurement of IL-8, a pro-inflammatory cytokine that attracts and activate neutrophils and acts as a mediator of angiogenesis^59^, showed that treatment with *S. salivarius* MS-oral-D6 did not induce increased inflammation, which was in clear contrast to *L. plantarum* WCFS1 and may be involved in the inability of the latter bacteria to significantly modulate re-epithelialization.

Proteins secreted by bacteria have been shown to play a prominent role in the modulation of re-epithelialization^9, 48–50^, and we could show that filter-sterilized conditioned medium of three *S. salivarius* strains used in this study consistently accelerated re-epithelialization. However, this effect appeared to depend on a larger sized (>7 kDa) secreted protein only present in the spent medium obtained from *S. salivarius* MS-oral-D6. Correspondingly, a high MW protein (>180 kDa) was only detected in the conditioned medium of *S. salivarius* MS-oral-D6 and not in that of strains MS-ileo-F1 and HSISS3. Proteomic analysis suggested that this protein could correspond to a secreted serine protease based on its apparent molecular weight as well as the diversity of the presence of its encoding gene in different strains of *S. salivarius*. Moreover, the involvement of this serine protease as the effector molecule in the observed stimulation of re-epithelialization kinetics is supported by the abolishment of the effect on wound healing by serine protease inhibitor (PMSF) pretreatment. The secreted serine protease of *S. salivarius* is a member of the subtilases superfamily characterized by a catalytic triad (Asp/Ser/His) similar to the one in trypsine serine proteases^60^. Notably, protease activity is essential for normal wound healing and is known to play a role in different phases of healing, including aiding detachment and migration of cells^61^. Taken together, the presence of the serine protease in the conditioned medium of *S. salivarius* MS-oral-D6 may explain the strain specific effects observed on re-epithelialization. However, a role of additional proteins, which may include the other proteins identified in the same strain’s conditioned medium cannot be excluded at this stage.

In conclusion, this study presents a high-throughput re-epithelialization model that enables the extraction of kinetic information regarding the capacity of bioactive substances to modulate this process. The model developed here uses the gingival cell line Ca9–22, but it is applicable to other cell lines as well (data not shown). Screening LAB for their effect in re-epithelialization led to the identification of *S. salivarius* as one of the most stimulatory species among the panel of organisms tested. In addition, *S. salivarius* MS-oral-D6 secreted serine proteases was identified as the most likely candidate effector molecule involved in enhanced re-epithelialization. It remains to be determined by what molecular mechanism this protein could modulate epithelial cell processes, including the acceleration of wound closure. Moreover, the possible contribution of other proteins produced and secreted by *S. salivarius* MS-oral-D6 to the accelerated re-epithelialization is currently not known and also deserves further study. Such mechanistic studies may open avenues to identify *S. salivarius* strains that produce the effector protein(s) in high amounts and could be considered as probiotics to support epithelial integrity in the oral cavity, or may enable strategies that involve purified forms of such protein(s) as a ‘bioactive’ in treatments with similar purposes. This study illustrates the importance of identification and mechanistic understanding of the involvement of microbial components in maintenance of oral health which can fuel innovative strategies to support oral health.

## Materials and Methods

### Epithelial cell line

Ca9–22 (JCRB0625) gingival epithelial cells were purchased from the National Institute of Biomedical Innovation JCRB Cell Bank (Osaka, JP). The cells were cultured in Dulbecco’s Modified Eagle Medium (DMEM) containing Glutamax (Gibco, Invitrogen, Paisley, UK) and supplemented with 10% fetal calf serum (FCS), 100 U/ml penicillin and 100 μg/ml streptomycin (Sigma-Aldrich, MO, USA). The cell line was cultured at 37 °C in a humidified atmosphere containing 5% CO_2_ and passaged every three to four days. Experiments were carried out between passages three to twenty.

### Bacterial strains and culture

The lactic acid bacteria (LAB) used in this study are listed in Table 2. The *Lactobacillus* strains and *Streptococcus mutans* NIZO1215 were obtained from the NIZO culture collection (NIZO food research, Ede, NL). The other streptococci were kindly provided by Dr. Tom van den Bogert from the Laboratory of Microbiology of the Wageningen University (Wageningen, NL). *Lactobacillus* strains were routinely cultured in de Man, Rogosa and Sharpe (MRS) medium (Merck Millipore, Darmstadt, DE) and *Streptococcus* strains were cultured in trypticase soy broth (TSB) (BD Difco, Le Pont de Claix, FR) supplemented with yeast extract (3 g/L). All lactic acid strains were grown at 37 °C with 5% CO_2_. *Porphyromonas gingivalis* W83 was kindly provided by Dr. Alexa M. G. A. Laheij from ACTA (Academic Centre for Dentistry Amsterdam, Amsterdam, NL) and grown anaerobically at 37 °C in Brain Heart Infusion (BHI) broth (BD Difco, Le Pont de Claix, FR) supplemented with haemin (5 mg/L) and menadione (1 mg/L). The GasPak™ EZ Anaerobe Container System Sachets (Becton, Dickinson and Company, NJ, USA) were used to produce an anaerobic environment inside of the containers.

**Table 2:** Lactic acid bacteria used in the screenings for their effect in oral re-epithelialization.

#	Species identification	Origin	Reference
1	*Lactobacillus acidophilus* NIZO221	Human	15
2	*Lactobacillus acidophilus* NIZO222	N/A	15
3	*Lactobacillus acidophilus* NIZO223	N/A	15
4	*Lactobacillus acidophilus* NIZO225	N/A	15
5	*Lactobacillus acidophilus* NIZO229	N/A	15
6	*Lactobacillus acidophilus* NIZO267	N/A	15
7	*Lactobacillus acidophilus* NIZO867	Human	15
8	*Lactobacillus casei* NIZO2928	Human saliva	15
9	*Lactobacillus casei* NIZO2929	Human saliva	15
10	*Lactobacillus fermentum* NIZO1220	N/A	15
11	*Lactobacillus fermentum* NIZO2517	Human oral cavity	15
12	*Lactobacillus fermentum* NIZO2930	Saliva	15
13	*Lactobacillus fermentum* NIZO2931	Human oral strain	15
14	*Lactobacillus fermentum* NIZO2932	Human saliva	15
15	*Lactobacillus fermentum* NIZO2933	Human saliva	15
16	*Lactobacillus fermentum* NIZO2934	Human saliva	15
17	*Lactobacillus fermentum* NIZO307	Human oral cavity	15
18	*Lactobacillus paracasei* NIZO2518	Child saliva	15
19	*Lactobacillus paracasei* NIZO2935	Oral source	15
20	*Lactobacillus paracasei* NIZO2936	Child saliva	15
21	*Lactobacillus paracasei* NIZO2945	Oral cavity	15
22	*Lactobacillus plantarum* NIZO2519	Human saliva	15
23	*Lactobacillus plantarum* WCFS1	Human saliva	71
24	*Lactobacillus rhamnosus* GG*	Human intestine	72
25	*Lactobacillus rhamnosus* NIZO2937	Human saliva	73
26	*Lactobacillus salivarius* NIZO2520	Saliva	15
27	*Lactobacillus salivarius* NIZO2521	Saliva	15
28	*Lactobacillus salivarius* NIZO2938	Human saliva	15
29	*Lactobacillus salivarius* NIZO2943	Human saliva	15
30	*Porphyromonas gingivalis* W83*	Human gingival sulcus	48
31	*Streptococcus bovis* HSISB1	Human ileostomy	74
32	*Streptococcus mitis* HSISM1	Human ileostomy	74
33	*Streptococcus mutans* NIZO1215	N/A	15
34	*Streptococcus salivarius* HSISS1	Human ileostomy	74
35	*Streptococcus salivarius* HSISS2	Human ileostomy	74
36	*Streptococcus salivarius* HSISS3	Human ileostomy	74
37	*Streptococcus salivarius* HSISS4	Human ileostomy	74
38	*Streptococcus salivarius* MS-ileo-F1	Human ileostomy	74
39	*Streptococcus salivarius* MS-oral-B3	Human oral cavity	74
40	*Streptococcus salivarius* MS-oral-D6	Human oral cavity	74

^*^ *P. gingivalis* and *L. rhamnosus* GG were included in this study for reference purposes. N/A not assigned.

### Preparation of bacterial treatments

Bacterial strains were grown until stationary phase, after which glycerol 15% (v/v) stocks were prepared and stored at −80 °C until used. On the day of the experiment, glycerol stocks were thawed and bacteria were pelleted by centrifugation (4,000 × g, 7 minutes at room temperature). The spent media were removed and the bacteria were re-suspended in FCS-free DMEM to obtain the original optical density at 600 nm (OD600). The samples were further diluted in FCS-free DMEM until the required multiplicity of infection (MOI). An OD600 of 1 corresponded to approximately 5 × 10^8^ CFU/ml. Scratch assays were performed using different MOIs. Initial screening efforts employed a standard MOI of 50, whereas subsequent experiments employed MOIs of 10, 50 and 250 to obtain dose-response curves.

### Conditioned medium collection and preparation

Bacterial strains were grown until stationary phase. Subsequently, the bacterial cells were removed by centrifugation (4,000 × g, 7 minutes at room temperature) to obtain the spent culture medium (conditioned medium) of which the pH was adjusted to 7.0 by addition of 1 M NaOH. Conditioned medium samples were filter-sterilized using a syringe filter with a pore size of 0.2 μm (Whatman, GE Healthcare Life Sciences, Dassel, DE) and tested for their capacity to promote re-epithelialization in comparison with the control medium (i.e., TSB medium in absence of bacteria). In order to remove small molecules and/or peptides as well as other bacterial growth medium constituents, the conditioned medium and control medium were passed through Zeba™ Spin Desalting columns (7 kDa cutoff, Thermo Scientific, Rockford, IL, USA). The desalted samples were divided in two equal volume aliquots, one of which was treated with 200 μg/ml proteinase K (1 hour at 37 °C) and the other was left untreated. Inactivation of proteinase K was accomplished by heating the samples to 98 °C for 10 minutes, followed by centrifugation (16,000 × g, 10 min at room temperature)^62^. After centrifugation, all samples were filtered sterilized (0.2 μm, Whatman, GE Healthcare Life Sciences) and then stored at −80 °C until used. Desalted-conditioned medium derived from *S. salivarius* MS-oral-D6 were treated with 1 mM phenylmethane sulfonyl fluoride (PMSF) for 1 hour at room temperature in order to inactivate the serine proteases present in the samples. After inactivation, the samples were stored at −80 °C until used.

### SDS-PAGE and silver staining

Conditioned medium proteins were precipitated with 10% (w/v) iced-cold trichloroacetic acid (TCA) in a 1:1 ratio and incubated for 30 minutes on ice. After incubation, the samples were centrifuged at 16,000 × g for 10 minutes at 4 °C. The supernatant was discarded and the protein pellet was washed twice with ice-cold acetone. The acetone was removed and the pellet was air-dried. Precipitated proteins were taken up in 2X Laemmli sample buffer^63^ with 1% β-mercaptoethanol and after boiling the samples, they were analysed by sodium dodecyl sulfate polyacrylamide gel electrophoresis (SDS-PAGE) using 4–15% Mini-POTEAN® TGX™ Precast Gels (Bio-Rad Laboratories, CA, USA). The SilverXpress® Silver Staining Kit (Thermo Scientific) was used to visualize the proteins in gel.

### Scratch Assay

Cells were seeded in tissue culture-treated 96-well plates (BD Falcon™, Corning, NY, USA) at a density of 3.5 × 10^4^ cells/well and grown overnight until confluence in DMEM supplemented with 10% FCS. The next day, cells were starved in FCS-free DMEM for 2 hours in order to decrease the basal levels of cell migration and proliferation that is induced by growth stimulatory components present in FCS. During the last 20 minutes of the starvation, the cellular cytoplasm were labelled with 2 μM CellTracker™ Red CMTPX (Molecular Probes, OR, USA) and the nuclei were stained with 2 μg/ml Hoechst 33342 (Molecular Probes). Following 2 hours starvation, the HTSScratcher (Peira, Antwerpen, BE) was used to make equally sized and reproducible longitudinal scratches (0.3 × 2 mm) in the cell monolayers of all the wells simultaneously. Detached cells and debris were removed by washing the cells twice with phosphate buffered saline (PBS). Subsequently, 100 μl of the bacterial treatments and controls in DMEM without FCS were added into the wells in a randomized manner. Conditioned medium was diluted in FCS-free DMEM in a 1:2 ratio. The positive control consisted of 4 ng/ml human transforming growth factor α (TGFα; R&D Systems, MN, USA), which acts as a mitogenic and motility factor. A combination of inhibitors of p38 (SB203580; Cell Signaling Technology, MA, USA) and MEK1/2 (U0126, Cell Signaling Technology) at a concentration of 10 μM each, served as negative control. Inhibition of p38 mitogen-activated protein kinase (MAPK) phosphorylation led to suppression of cell migration^64, 65^ and inhibition of MEK1/2 phosphorylation blocked the activation of ERK1/2 MAPK cascade that mediates cell proliferation and survival^66^. FCS-free DMEM was used as non-treated control. The overall quality of each run of the 96-well based assay was assessed by calculation of the Z’ factor, which is defined by the means and standard deviations of both the positive and negative controls. Values between 0.5 and 1 indicated an excellent assay; values between 0 and 0.5 were considered acceptable; and assays with negative values were discarded^31^.

### Automated image acquisition and segmentation

Live microscopy images were acquired using the BD Pathway 855 Bioimaging System (BD Biosciences, CA, USA) under controlled temperature and atmosphere (37 °C and 5% CO_2_). Hoechst 33342 and CellTracker™ Red CMTPX images were acquired using an excitation filter of 350 nm and 577 nm, respectively. Bright-field images served as visual control for cell morphology and vitality. The microscope was programmed to acquire fluorescent and bright-field images of the same field of each well every 20 minutes for 5 hours using a 4x objective. Image segmentation was carried out using CellProfiler 2.1.1 (http://www.cellprofiler.org/). Two segmentation pipelines were established: a tissue-recognition pipeline and a cell-recognition pipeline. The tissue-recognition pipeline was designed to identify the number of pixels of the scratched area that were covered by the monolayer of cells (tissue) in consecutive images. The number of pixels constituting the scratch was calculated by subtracting the pixels covered by cells from the total number of pixels in the image. The percentage of scratch closure in consecutive images was then calculated as 1 − [(# pixels in the scratch at time x)/(# pixels in the scratch at time 0) * 100]. The cell-recognition pipeline was designed to identify single cells in sequential images and record their location as well as other features such as shape, size and fluorochrome intensity. The features extracted by the cell-recognition pipeline were then used to model the kinetics of the re-epithelialization process.

### Modelling of the kinetics of oral re-epithelialization

FCS Express 4 Plus (De Novo Software, CA, USA) was used to visualize the features extracted by the cell-recognition pipeline established in CellProfiler. A scatterplot was created with the location (x, y) of the cells within a well at the starting point of the assay. A gate was placed on the scratched area and a batch process was setup for each well to record the number of cells infiltrating the wound throughout time. The lag time (λ in minutes), repair rate (μ_m_ in cells minute^−1^) and the maximum number of cells (A) inside of the scratched area of each well were calculated in R (http://www.R-project.org/)^67^ by a nonlinear least squares regression of the modified Gompertz function^30^ using the Levenberg-Marquardt algorithm^68^. The Levenberg-Marquardt algorithm reduces the sum of the squared errors between the modelled and the measured data points by iteratively improving the parameter values^69^. The μ_m_ and A parameter values of each treatment were normalized against the corresponding average values of the non-treated control to obtain the performance value (μ_m_*A). When appropriate (i.e., n ≥ 3), replicate averages and standard error of the mean (SEM) for each treatment were calculated and significant differences from the non-treated control were determined using GraphPad Prism version 6.00 (GraphPad Software, Inc, CA, USA) with a limit of significance set at *P* < 0.05. To assess the relation between the A parameter and the relative of scratch closure, average values were normalized against the non-treated control and their relation was assessed in R using Spearman Rank Correlation.

### Quantification of lactate, acetate and IL-8 in cell culture supernatants

Fermentation end products (i.e., lactic acid and acetic acid) of LAB during the 5 hours re-epithelialization assay were determined in cell culture supernatant collected at the end of the 5 hours re-epithelialization assay by nuclear magnetic resonance (NMR), using an internal standard (0.5 mM) to calculate the absolute concentrations of both acids in each sample. The potential correlation of the metabolite concentrations with the observed wound repair kinetics observed in the re-epithelialization assay was assessed in R using Pearson Correlation analysis. The ELISA Ready SET Go! Kit (BD Biosciences, CA, USA) was used to determine the amount of interleukin-8 (IL-8) in the supernatants of cells exposed to selected bacterial treatments (MOI 250) during the 5 hours re-epithelialization assay.

### In-gel trypsin digestion and LC-MS/MS

The proteins contained in the conditioned medium of *Streptococcus salivarius* MS-oral-D6 were precipitated with 10% (w/v) iced-cold trichloroacetic acid (TCA) and separated by SDS-PAGE as described in Materials and Methods. Ready-to-use Coomassie protein stain InstantBlue (Expedeon, Cambridgeshire, UK) was used to visualize the proteins on gel. In-gel trypsin digestion followed by liquid-chromatography tadem mass spectrometry (LC-MS/MS) was performed as previously described^70^. The protein band (>180 kDa) was excised from the gel and transferred to a low binding protein microcentrifuge tube. A blank gel slice was used as control. The gel pieces were washed twice with water and disulfide bridges were then reduced with 10 mM dithiotreitol (DTT) in 50 mM ammonium bicarbonate (ABC, pH 8.0) for one hour at 60 °C. Subsequently, the DTT is replaced by 15 mM iodoacetamide in in 50 mM Tris buffer pH 8.0 and incubated at room temperature in the dark for one hour. After washing twice with ABC, the gel pieces were incubated overnight at room temperature in ABC containing 5 ng/μl bovine sequencing grade trypsin (Roche). The samples were sonicated briefly in an ultrasonic water bath to extract the digested peptides after which 10% trifluoroacetic acid (TFA) was added to adjust the pH between 2 and 4. To assure gel-free samples, the peptide solutions were desalted using C18 stage-tip columns made in-house. The columns were washed twice with methanol and equilibrated with formic acid (1:1000). Peptide solutions were added to the columns and eluted with a vacuum manifold. The columns were then washed with formic acid and transferred to low binding protein microcentrifuge tubes. Peptides were eluted with solution of 50% acetonitrile and 50% formic acid (1:1000) using a 10-ml plastic syringe. The LC-MS/MS analysis was performed on a Proxeon EASY-nLC system (Thermo Scientific) coupled with an LTQ-Orbitrap XL mass spectrometer (Thermo Scientific). For protein identification and relative quantitation, MaxQuant 1.3.0.5 software package was used with false discovery rates of 1% both on peptide and protein level after matching the MS/MS spectra against a common contaminants database and *S. salivarius* database.

### Data availability

The datasets generated during the current study are available from the corresponding author on reasonable request.

## Electronic supplementary material

Supplementary Information
