# Supplementary material for: Streptococcus salivarius MS-oral-D6 promotes gingival re-epithelialization in vitro through a secreted serine protease
Source: Sci Rep. 2017 Sep 11;7:11100. doi: 10.1038/s41598-017-11446-z (PMC5593969; doi:10.1038/s41598-017-11446-z)
Supplement: Supplementary file 1 — Supplementary Information [file 41598_2017_11446_MOESM1_ESM.pdf]

Supplementary Information

Streptococcus salivarius MS-oral-D6 promotes gingival re-epithelialization *in vitro* through a secreted serine protease

Marcela M. Fernandez-Gutierrez[1,2], Peter P. J. Roosjen[3], Eveline Ultee[2], Maarten Agelink[2], Jacques J.M. Vervoort[4], Bart Keijser[1,5,6], Jerry M. Wells[2], Michiel Kleerebezem[1,2*]

[1] TI Food and Nutrition, Nieuwe Kanaal 9-A, 6709 PA, Wageningen, The Netherlands.

[2]Host-Microbe Interactomics Group, Department of Animal Sciences, Wageningen University & Research, De Elst 1, 6708 WD, Wageningen, The Netherlands.

[3]Laboratory of Geo-Information Science and Remote Sensing, Wageningen University & Research, Droevendaalsesteeg 3, 6708 PB, Wageningen, The Netherlands.

[4] Biochemistry Group, Department of Agrotechnology and Food Sciences, Wageningen University & Research, Stippeneng 4, 6708 WE, Wageningen, The Netherlands.

[5] TNO Microbiology and Systems Biology, Utrechtseweg 48, 3704 HE, Zeist, The Netherlands.

[6]Department of Preventive Dentistry, Academic Centre for Dentistry Amsterdam, University of Amsterdam and Vrije Universiteit Amsterdam, Gustav Mahlerlaan 3004, 1081 LA, Amsterdam, The Netherlands.

***Correspondence:**

Michiel Kleerebezem

Host-Microbe Interactomics, Wageningen University, De Elst 1, 6708 WD, Wageningen, The Netherlands

Tel: +31 317 483822

Email: michiel.kleerebezem@wur.nl

Supplementary Figures

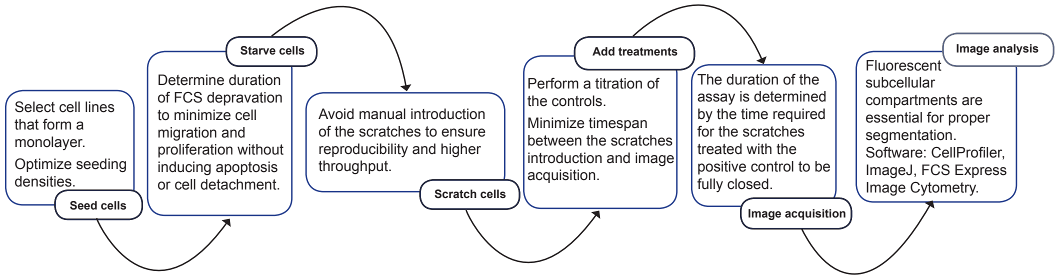

Figure S1. Scratch assay workflow. The assay workflow and crucial considerations are indicated in each step.

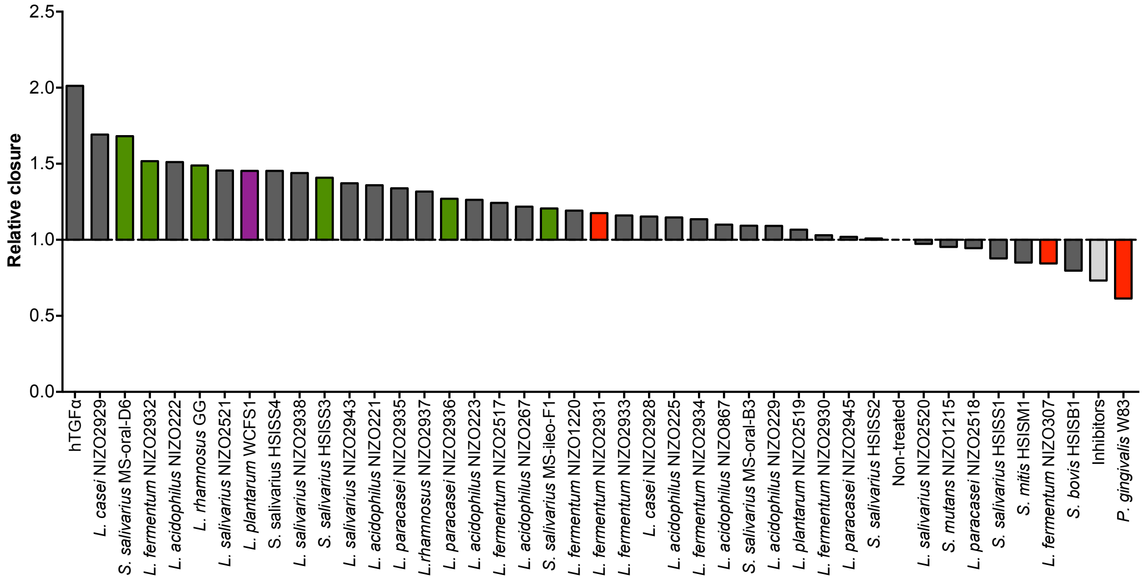

Figure S2. Relative scratch closure after exposure to different bacterial treatments. The closure of the scratches after stimulation of Ca9-22 cells with the bacterial treatments was calculated relative to that of the non-treated control. Green bars: potential stimulators; red bars: potential attenuators; purple bar: minor modulator. Human transforming growth factor alpha (hTGF, 4 ng/ml) and a combination of p38 (SB203580, 10 µM) and MEK1/2 (U0126, 10 µM) inhibitors were used as positive and negative controls respectively.

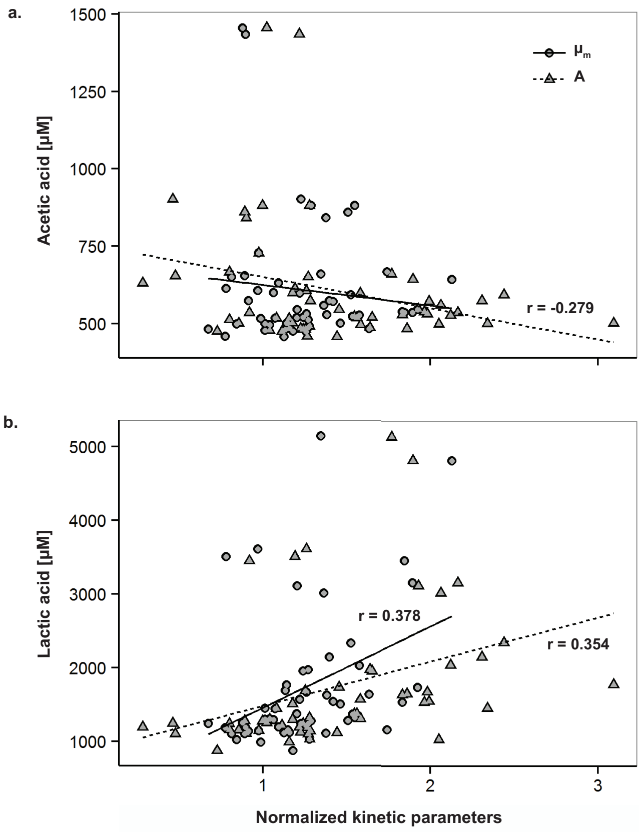

Figure S3. Correlation between metabolic end products of bacteria and re-epithelialization kinetics. Association between the concentrations of (a) acetic and (b) lactic acid with the kinetic parameters (A and μ_m) of re-epithelialization assessed by Pearson correlation (n = 54).

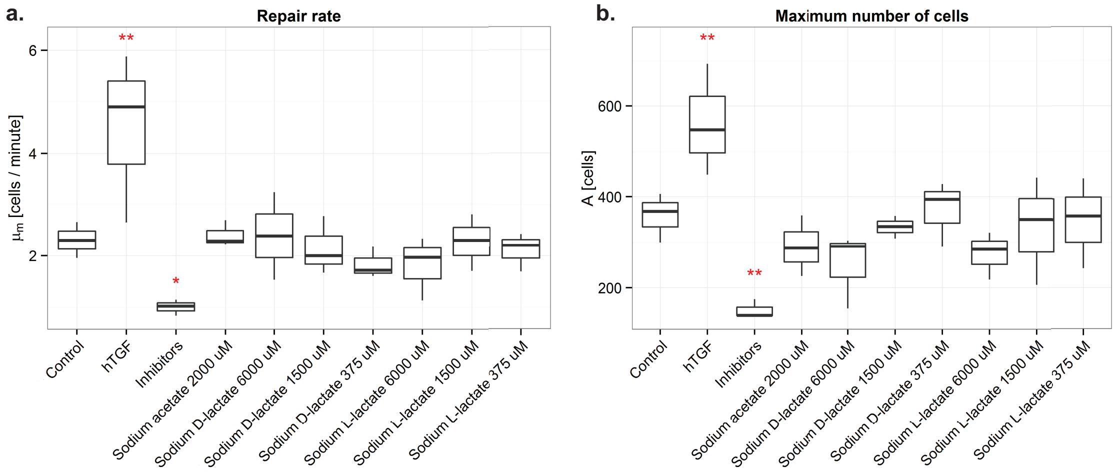

Figure S4. Re-epithelialization kinetics of cells exposed to acetate, D-lactate and L-lactate. (a) Repair rate. (b) Maximum number of cells within the scratched area after 5 hours.

Table S1. Re-epithelialization parameter values obtained from the preliminary screening. Estimated parameters (μ_m, λ, A) and goodness of fit (R^2) obtained after fitting the Gompertz function through the enumeration of infiltrating cells over time.

Treatment	μ_m	SEM_μ_m	λ	SEM_λ	A	SEM_A	RMSE	R^2
Non-treated	2.063	0.222	-1.705	9.229	373.044	16.060	19.489	0.973
Non-treated	3.387	0.088	27.853	1.542	396.658	2.604	4.742	0.999
Non-treated	3.279	0.098	35.843	2.116	483.291	5.235	6.837	0.999
Non-treated	3.141	0.187	-1.647	4.876	533.865	11.488	15.415	0.992
hTGFα	6.758	0.398	2.752	3.418	757.562	9.661	20.526	0.993
hTGFα	6.357	0.390	4.831	3.850	781.560	11.610	22.570	0.993
hTGFα	12.769	1.106	25.065	2.927	802.766	9.705	25.753	0.992
hTGFα	11.183	0.922	17.142	3.331	847.313	11.089	28.153	0.990
Inhibitors	1.005	0.138	1.545	10.916	166.001	8.021	11.053	0.959
Inhibitors	2.244	0.151	5.285	2.467	153.482	1.459	3.983	0.993
Inhibitors	1.942	0.096	32.367	2.852	220.714	2.711	4.964	0.996
Inhibitors	2.702	0.090	13.794	1.841	286.696	2.055	4.357	0.998
L. acidophilus NIZO221	2.202	0.153	-5.555	6.030	402.460	11.038	13.451	0.988
L. acidophilus NIZO221	4.684	0.268	5.744	4.251	709.642	12.944	19.716	0.993
L. acidophilus NIZO222	4.048	0.298	14.540	5.223	583.356	13.409	20.769	0.990
L. acidophilus NIZO222	5.546	0.340	18.293	2.718	464.329	4.932	11.914	0.995
L. acidophilus NIZO223	4.044	0.253	7.086	3.766	474.305	6.887	13.844	0.993
L. acidophilus NIZO223	3.726	0.184	7.507	3.606	551.548	8.509	13.240	0.995
L. acidophilus NIZO225	2.388	0.187	-5.197	6.776	436.145	13.482	16.409	0.986
L. acidophilus NIZO225	3.854	0.230	3.894	4.163	538.170	9.048	15.429	0.993
L. acidophilus NIZO229	3.767	0.326	-2.556	6.345	553.970	13.915	22.977	0.983
L. acidophilus NIZO229	2.816	0.219	1.524	6.060	450.367	11.786	17.029	0.987
L. acidophilus NIZO267	2.884	0.222	-8.883	7.369	603.444	22.685	22.026	0.985
L. acidophilus NIZO267	3.317	0.237	-4.034	5.339	497.914	10.522	17.046	0.988
L. acidophilus NIZO867	3.566	0.353	-4.087	6.740	477.394	12.031	22.299	0.978
L. acidophilus NIZO867	4.367	0.305	-0.739	4.232	513.221	8.004	16.578	0.990
L. casei NIZO2928	2.349	0.099	20.401	2.603	288.089	3.135	5.666	0.997
L. casei NIZO2928	4.172	0.188	14.882	2.543	455.897	4.560	9.405	0.996
L. casei NIZO2929	4.456	0.162	10.244	2.495	613.671	6.392	10.728	0.997
L. casei NIZO2929	4.102	0.233	8.360	3.868	558.495	8.866	15.228	0.994
L. fermentum NIZO1220	3.478	0.158	11.404	3.430	540.528	8.426	11.990	0.996
L. fermentum NIZO1220	3.694	0.255	-4.808	5.104	547.553	10.909	18.023	0.989
L. fermentum NIZO2517	4.367	0.271	8.418	3.420	462.678	6.019	13.060	0.993
L. fermentum NIZO2517	3.965	0.196	10.395	3.340	534.176	7.373	12.686	0.995
L. fermentum NIZO2930	3.034	0.223	-1.071	6.213	538.680	15.426	19.238	0.987
L. fermentum NIZO2930	3.858	0.143	11.431	2.245	458.286	4.070	7.932	0.997
L. fermentum NIZO2931	2.855	0.207	15.438	5.399	439.657	11.008	15.483	0.990
L. fermentum NIZO2931	2.200	0.206	-6.566	8.311	414.243	15.973	18.598	0.979
L. fermentum NIZO2932	4.703	0.354	7.576	4.850	598.438	11.513	21.365	0.989

L. fermentum NIZO2932	4.431	0.198	12.777	2.945	582.075	7.096	12.405	0.996
L. fermentum NIZO2933	3.382	0.238	11.479	5.377	535.058	13.260	18.378	0.990
L. fermentum NIZO2933	3.288	0.245	1.827	5.134	449.916	9.110	16.059	0.988
L. fermentum NIZO2934	3.578	0.306	-3.048	6.062	504.246	11.787	20.542	0.984
L. fermentum NIZO2934	2.021	0.181	-9.869	7.565	351.634	11.102	14.991	0.981
L. fermentum NIZO307	1.909	0.193	-4.612	7.800	299.344	9.452	14.491	0.977
L. fermentum NIZO307	3.365	0.255	4.495	4.566	394.486	6.847	13.920	0.989
L. paracasei NIZO2518	2.951	0.149	11.733	3.984	486.020	9.243	12.033	0.995
L. paracasei NIZO2518	2.695	0.300	-11.068	8.910	435.511	15.107	22.893	0.971
L. paracasei NIZO2935	2.984	0.140	14.742	3.263	421.068	5.975	9.496	0.996
L. paracasei NIZO2935	2.956	0.225	0.404	6.429	525.502	15.794	19.486	0.987
L. paracasei NIZO2936	4.664	0.333	6.884	4.388	559.395	9.511	18.753	0.990
L. paracasei NIZO2936	5.237	0.281	3.572	3.200	604.986	7.298	15.081	0.994
L. paracasei NIZO2945	2.020	0.197	-11.301	8.221	350.321	11.820	16.150	0.977
L. paracasei NIZO2945	4.291	0.327	1.973	4.622	504.729	8.746	17.912	0.988
L. plantarum NIZO2519	5.781	0.307	6.491	2.383	488.376	4.373	10.937	0.995
L. plantarum NIZO2519	3.779	0.275	-0.584	5.666	603.011	14.480	21.230	0.988
L. plantarum WCFS1	3.870	0.340	10.364	5.778	505.312	11.892	21.186	0.985
L. plantarum WCFS1	3.851	0.282	3.946	5.052	529.654	10.730	18.617	0.989
L. rhamnosus GG	5.115	0.336	2.611	4.214	644.435	10.420	19.977	0.991
L. rhamnosus GG	3.807	0.206	13.318	3.665	517.701	8.015	13.430	0.995
L. rhamnosus NIZO2937	2.576	0.151	16.406	4.352	394.780	7.990	11.248	0.993
L. rhamnosus NIZO2937	3.240	0.269	11.024	5.931	469.145	12.014	18.869	0.986
L. salivarius NIZO2520	2.871	0.163	3.814	4.867	524.758	12.629	14.560	0.993
L. salivarius NIZO2520	4.735	0.334	3.665	3.763	482.040	6.723	15.273	0.990
L. salivarius NIZO2521	2.236	0.190	13.953	6.274	339.745	9.693	14.019	0.986
L. salivarius NIZO2521	4.776	0.350	1.970	4.745	606.738	11.032	21.034	0.989
L. salivarius NIZO2938	4.117	0.253	5.968	4.937	692.678	15.925	20.778	0.992
L. salivarius NIZO2938	3.796	0.251	8.229	4.650	537.134	10.452	17.157	0.991
L. salivarius NIZO2943	5.505	0.910	8.431	6.017	373.175	8.753	23.754	0.959
L. salivarius NIZO2943	1.489	0.197	-12.067	12.057	286.851	15.560	17.918	0.957
P. gingivalis W83	3.886	0.268	1.745	3.233	343.523	4.085	10.138	0.991
P. gingivalis W83	4.017	0.260	13.458	2.741	319.152	3.380	8.504	0.993
P. gingivalis W83	3.857	0.380	20.001	3.873	284.127	4.375	11.137	0.987
P. gingivalis W83	2.208	0.212	-0.592	3.639	155.689	2.135	5.860	0.983
S. bovis HSISB1	2.658	0.212	-2.583	5.328	347.927	6.954	13.112	0.986
S. bovis HSISB1	2.428	0.161	-4.680	5.386	405.702	9.261	12.962	0.990
S. mitis HSISM1	3.007	0.268	-0.414	6.680	455.614	12.476	19.619	0.983
S. mitis HSISM1	4.852	0.230	10.980	2.925	586.425	6.806	13.078	0.996
S. mutans NIZO1215	2.456	0.280	-1.911	7.780	330.646	9.805	17.897	0.972
S. mutans NIZO1215	2.728	0.166	-5.230	4.546	410.563	7.324	11.891	0.991
S. salivarius HSISS1	2.562	0.165	-0.300	4.766	383.315	7.435	11.886	0.991
S. salivarius HSISS1	2.496	0.239	-12.955	8.164	436.620	14.495	19.691	0.978
S. salivarius HSISS2	2.794	0.162	5.061	4.356	430.180	8.089	12.096	0.993

S. salivarius HSISS2	3.552	0.200	-1.664	4.415	571.413	10.662	15.536	0.993
S. salivarius HSISS3	3.765	0.320	-8.290	6.604	590.106	15.259	23.816	0.983
S. salivarius HSISS3	3.951	0.249	3.196	4.133	510.647	8.200	15.284	0.992
S. salivarius HSISS4	3.036	0.167	1.082	4.539	526.667	10.990	14.060	0.993
S. salivarius HSISS4	2.676	0.233	-15.673	9.055	623.015	30.786	25.030	0.980
S. salivarius MS-ileo-F1	4.238	0.149	13.609	2.729	686.391	8.935	11.797	0.998
S. salivarius MS-ileo-F1	2.761	0.167	14.701	5.259	525.992	15.674	15.573	0.992
S. salivarius MS-oral-B3	3.089	0.138	17.773	3.442	497.342	8.364	10.844	0.996
S. salivarius MS-oral-B3	2.960	0.183	4.652	4.461	430.211	7.964	12.881	0.992
S. salivarius MS-oral-D6	4.710	0.357	5.496	5.878	753.939	19.739	27.863	0.988
S. salivarius MS-oral-D6	5.018	0.228	12.373	3.065	678.156	8.703	14.765	0.996

Table S2. Lactic and acetic acid quantification. The concentration of accumulated lactic and acetic acid in the supernatant of Ca9-22 cells treated with lactic acid bacteria for 5 hours.

Sample	MOI	Average lactic acid (µM)	SD	Average acetic acid (µM)	SD
S. salivarius MS-ileo-F1	10	1132.70	109.26	489.66	8.48
S. salivarius MS-ileo-F1	50	1806.30	166.35	484.83	1.74
S. salivarius MS-ileo-F1	250	3297.59	150.40	534.78	0.08
S. salivarius HSISS3	10	1409.29	39.12	510.94	11.65
S. salivarius HSISS3	50	1854.83	480.18	555.26	36.59
S. salivarius HSISS3	250	1636.28	95.47	558.10	13.16
L. paracasei NIZO2936	10	1555.87	111.50	524.13	6.90
L. paracasei NIZO2936	50	2085.80	55.07	549.75	23.58
L. paracasei NIZO2936	250	4972.71	167.86	650.92	8.54
L. plantarum WCFS1	10	1467.63	160.46	511.91	16.32
L. plantarum WCFS1	50	1859.18	92.97	510.18	9.73
L. plantarum WCFS1	250	3058.46	49.31	551.83	6.80
S. salivarius MS oral D6	10	1088.16	98.32	487.01	28.41
S. salivarius MS oral D6	50	1119.90	2.27	470.87	13.56
S. salivarius MS oral D6	250	1548.31	21.41	567.48	30.34
L. fermentum NIZO1220	10	1135.79	105.26	501.61	10.37
L. fermentum NIZO1220	50	1217.28	77.94	586.34	12.89
L. fermentum NIZO1220	250	1299.28	20.45	870.57	10.60
L. rhamnosus GG	10	1256.33	41.33	475.82	1.76
L. rhamnosus GG	50	1597.45	89.39	490.92	10.09
L. rhamnosus GG	250	3557.57	51.46	609.40	3.86
P. gingivalis W83	10	1014.32	139.60	487.59	13.05
P. gingivalis W83	50	1130.31	27.70	659.93	6.20
P. gingivalis W83	250	1218.68	25.37	766.21	135.26
L. fermentum NIZO307	10	1123.77	22.25	689.05	38.72
L. fermentum NIZO307	50	1191.20	83.64	861.03	19.83
L. fermentum NIZO307	250	1221.19	30.63	1444.98	10.23

Table S3. Conservation of the identified secreted proteins among *S. salivarius* sequenced genomes. Presence (+) or absence (-) of the proteins identified in the spent culture medium of *Streptococcus salivarius* MS-oral-D6 among the sequenced strains of the same species.

Strain	Serine protease	Peptidase M26	Peptidoglycan hydrolase	Surface antigen
SK126	-	-	+	+
726_SSAL	-	-	+	-
39-09 S16	-	-	+	+
ATCC 7073	-	-	+	+
HSISS2	-	-	+	+
NCTC 8618	-	+	+	+
JIM8777	-	+	+	+
CCHSS3	-	+	+	+
57.I	-	+	+	+
JF	-	+	+	+
PS4	-	+	+	+
K12	-	+	+	+
20-02 S1	-	+	-	+
22-08 S7	-	+	+	+
34-19 S9	-	+	+	+
34-24 S10	-	+	+	+
84-12 S20	-	+	+	+
37-09 S13	-	+	-	+
39-07 S15	-	+	+	-
40-02 S18	-	+	+	+
85-04 S22	-	+	+	+
85-05 S23	-	+	+	+
140_SSAL	-	+	+	+
2202 S3	-	+	+	+
HSISS3	-	+	+	+
GED7778A	+	+	+	+
M18	+	-	+	+
1003_SOLI	+	-	+	+
HSISS4	+	+	+	+
NU10	+	+	+	+
YU10	+	+	+	+
UC3162	+	+	+	+
KB005	+	+	+	+
1270_SSAL	+	+	+	+
20-12 S2	+	+	-	+
37-08 S12	+	+	+	-
HSISS1	+	+	+	+
